# Optimization system for training efficiency and load balance based on the fusion of heart rate and inertial sensors

**DOI:** 10.1016/j.pmedr.2024.102710

**Published:** 2024-03-29

**Authors:** Chen Wang, Man Tang, Kun Xiao, Defa Wang, Bin Li

**Affiliations:** a Head of Higher-educational Engineering Research Centre for Intelligence and Automation in Construction of Fujian Province, College of Civil Engineering, Huaqiao University, 361021 Xiamen, China; b Higher-educational Engineering Research Centre for Intelligence and Automation in Construction of Fujian Province, College of Civil Engineering, Huaqiao University, 361021 Xiamen, China; c Department of Physical Education, Xiamen Institute of Technology, Xiamen 361021, China; d China Railway No.18 Bureau Group No.1 Engineering Co., Ltd, 072750, Zhuozhou District, Baoding City, Hebei Province, China

**Keywords:** Training efficiency and load, Inertial sensors, Random forest algorithm, Physiological fatigue, Balance optimization system

## Abstract

**Objectives:**

To enhance the daily training quality of athletes without inducing significant physiological fatigue, aiming to achieve a balance between training efficiency and load.

**Design methods:**

Firstly, we developed an activity classification training model using the random forest algorithm and introduced the “effective training rate” (the ratio of effective activity time to total time) as a metric for assessing athlete training efficiency. Secondly, a method for rating athlete training load was established, involving qualitative and quantitative analyses of physiological fatigue through subjective fatigue scores and heart rate data. Lastly, an optimization system for training efficiency and load balance, utilizing multiple inertial sensors, was created. Athlete states were categorized into nine types based on the training load and efficiency ratings, with corresponding management recommendations provided.

**Results:**

Overall, this study, combining a sports activity recognition model with a physiological fatigue assessment model, has developed a training efficiency and load balance optimization system with excellent performance. The results indicate that the prediction accuracy of the sports activity recognition model is as high as 94.70%. Additionally, the physiological fatigue assessment model, utilizing average relative heart rate and average RPE score as evaluation metrics, demonstrates a good overall fit, validating the feasibility of this model.

**Conclusions:**

This study, based on relative heart rate and wearable devices to monitor athlete physiological fatigue, has developed a balanced optimization system for training efficiency and load. It provides a reference for athletes’ physical health and fatigue levels, offering corresponding management recommendations for coaches and relevant professionals.

## 1 Introduction

With the improvement of people’s living standards and the diversified development of spiritual life, sports and athletic activities have garnered increased attention. Since the 1960 s, the field of sports training has seen the emergence of various research perspectives, with a crucial focus on in-depth studies of the changes in athletes’ physical capabilities under intense exercise stimuli (Cui et al., 2023). Consequently, several scholars have proposed the theory of supercompensation, suggesting that during the procecss of fatigue recovery, body structure, and functional rebuilding after appropriate training, not only recovery but also surpassing the original level can occur (Maciejczyk et al., 2014, Bompa and Haff, 2009). However, the quantification of appropriate training has not been determined, necessitating the identification of a balance point between athletes’ physiological function and training efficiency. Moreover, with the increasing intensity of competition, many athletes often experience sports fatigue or even injuries due to inappropriate exercise during daily training or competitions (Wang, 2022). This inconvenience significantly affects their learning, health, and daily life. In severe cases, it can also impact their routine training, consequently affecting their performance in competitions. Therefore, real-time monitoring of athletes’ training status is essential to prevent injuries while maximizing training efficiency.

With technological advancements, wearable sensors can monitor human movement information, vital signs, and environmental information. Combined with relevant theories of data analysis, specific feedback recommendations can be provided (Fang and Chang, 2016, Lyons et al., 2005). The identification of human body postures through wearable devices has become feasible. However, for human activity recognition, single or individual types of sensors have certain limitations (Mathie et al., 2003, Cheng and Jhan, 2012, Ohnishi et al., 2019, Catal et al., 2015). Previous studies have mainly focused on coarse-grained activities such as walking, running, and sitting (Qiu et al., 2022), with relatively fewer studies on complex sports activities. In the field of motion recognition, the primary advantage of multi-sensor fusion methods over single sensors is their ability to provide richer limb movement information, thereby improving the accuracy of subsequent recognition algorithms. Therefore, the application of multiple types of wearable sensors can achieve high-precision recognition of human activities and states, enabling these devices to handle complex sports movements with increased robustness. Consequently, wearable sensors play a crucial role in sports activity recognition (Feng et al., 2023).

As of now, a fully mature fatigue monitoring system has not yet been developed (Khan et al., 2016). In previous studies, physiological indicators were often singularly chosen as effective measures of post-exercise fatigue response. However, due to significant variations in many physiological indicators during physical activity, selecting these indicators to predict physical fatigue states is challenging. Subsequent advancements in the research methods for studying physical fatigue categorized them into subjective self-reports and objective performance measurements (Zhu et al., 2022). Subjective fatigue measurements can efficiently assess the degree of fatigue in the body, for example, using the Borg CR10 scale for Rating of Perceived Exertion (RPE). This is commonly employed as a qualitative and cost-effective method to evaluate the training load on athletes. Therefore, considering both these objective biomarkers and subjective indicators of training load, such as perceived exertion ratings, allows for a more precise quantification of training load and its perception. This integrated approach contributes to a comprehensive understanding of the physiological and subjective states of athletes, providing more comprehensive guidance for optimizing training plans.

Given the above, this study addresses the susceptibility of sports training to injuries by developing a Training Efficiency and Load Balancing Optimization System based on the fusion of multiple wearable sensors. Firstly, the research establishes an activity recognition model based on the fusion of various types of inertial sensors. Utilizing the Random Forest algorithm, an activity classification training model is developed and validated for effectiveness using the leave-one-out method. The study then introduces the “Effective Training Rate” as an evaluation metric and establishes a rating method for assessing athletes’ training efficiency. Secondly, a rating method for athletes’ training load is established. Qualitative and quantitative analyses of physiological fatigue are conducted through subjective fatigue quantification scores and heart rate data. The scientific and accurate evaluation of construction workers’ physiological fatigue based on the relative heart rate demonstrates the validity of this physiological indicator. Finally, a Construction Efficiency Load Balancing Optimization System is developed based on the fusion of multiple inertial sensors. Athletes’ states are categorized into nine types according to the methods for evaluating training load and efficiency. The results of athlete state ratings can provide corresponding management recommendations for coaches and relevant professionals.

## 2 The latest development of physiological fatigue and load management in exercise training

### 2.1 Research on the application of activity recognition technology based on wearable devices

In recent years, activity recognition technology based on wearable sensors has gained popularity in the field of human activity recognition. It has also been widely applied in various domains, including healthcare (Serrano et al., 2017, Memar et al., 2018, Memar et al., 2017, Uddin, 2019), smart homes, and sports tracking (Ride et al., 2013, Stamm et al., 2011). These wearable sensors, combined with machine learning techniques, not only accurately predict human activities but also monitor individuals’ physiological indicators and movement states in real-time. For instance, Sungho Suh et al. (Suh et al., 2023), considering the direction of a single sensor as well as spatial and temporal features, proposed a novel adversarial learning framework based on Transformer, named TASKED, to identify human activities using wearable sensors. Naima Qamar et al. (Qamar et al., 2020), based on wearable accelerometer sensors, presented a feature extraction framework effective in recognizing human activities independent of sensor placement.

However, the capability of a single or a single type of sensor for recognizing complex activities is limited. Scholars have thus started to employ multiple sensors for data fusion to achieve higher activity recognition rates. For example, Wang et al. (Wang et al., 2016) explored the ability of the built-in triaxial accelerometer and gyroscope in smartphones to identify human body activities. The experiments showed that the fusion of accelerometer and gyroscope data contributed to better recognition performance compared to using a single data source. Qin and Zhuo (Ni et al., 2020), based on accelerometers and gyroscopes, employed a stacked denoising autoencoder (SDAE) for deep learning, achieving the recognition and classification of three types of daily activities. Gu (Gu et al., 2018) validated the combination of sensor data from multiple sensors (accelerometer, gyroscope, magnetometer, and barometer), obtaining higher recognition accuracy compared to using only accelerometer sensor data. Ye et al. (Chen and Wang, 2017) collected data from five inertial sensors, proposed a hierarchical recognition method based on artificial neural networks, and effectively identified concurrent and cross activities.

In the past, scholars mainly focused on the application of human activity and status recognition in fields such as health, healthcare, and daily activities. Recently, more research has begun to apply human activity and status recognition to the field of sports. Wael Y. Alghamdi (Alghamdi, 2023) proposed a new technology using wearable technology and recurrent neural networks to predict the health status of soccer players. The system can monitor the health status of athletes in real-time, making it one of the first applications of wearable sensors in athletes’ physical fitness and health. Wilk, Mariusz P. et al. (Wilk et al., 2020), utilizing the complementary characteristics of visual and inertial sensor modalities, combined with a novel multimodal sensor fusion algorithm, achieved computationally efficient three-dimensional (3-D) pose detection of wearable devices. By fusing information from two external reference points captured by a camera with the orientation captured by inertial motion sensors, three-dimensional posture can be determined, and it can be well applied to tracking barbell squat movements. Liu et al. (Liu et al., 2020) used multiple miniature inertial sensor nodes to collect the motion of kayakers. In the studies mentioned above, the high-precision recognition of sports activities using multiple sensors has been well demonstrated. Therefore, these technologies can be effectively utilized to assist coaches in athlete management and training supervision.

### 2.2 Athletes training load monitoring management research

In the past fifteen years, the quantification and monitoring of training loads have been the subject of much scientific work (Djaoui et al., 2017, Thorpe et al., 2017). In order to gain an understanding of the training load and its effect on the athlete, a number of potential markers are available for use. However, very few of these markers have strong scientific evidence supporting their use, and there is yet to be a single, definitive marker described in the literature (Halson, 2014).

When monitoring training load, the load units can be thought of as either external or internal (Impellizzeri et al., 2019). Traditionally, external load has been the foundation of most monitoring systems. External load is defined as the work completed by the athlete, measured independently of his or her internal characteristics (Wallace et al., 2009). In the sport of cycling, power output-measuring devices such as SRM™ and PowerTap™ allow the continuous measurement of work rate (power output) (Jobson et al., 2009). Training and competition can be recorded and data can be analyzed to provide information on a number of parameters. In team sports, time–motion analysis (TMA), including global positioning system (GPS) tracking and movement pattern analysis via digital video (such as ProZone™) are becoming increasingly popular to monitor athletes (Taylor et al., 2012). In addition, measurements of neuromuscular function, such as jump tests, sprint performance, as well as isometric and isotonic force assessments, are commonly employed in team sports environments (Twist and Highton, 2013).

Regarding internal load, the simplest and most cost-effective monitoring methods primarily involve questionnaire surveys. For instance, Vacher, P. et al. (Vacher et al., 2019) assessed the psychological states of 12 national swimmers using the RESTQ-1-R Sports Questionnaire and the Emotions and Mood States Questionnaire. However, this approach lacks real-time data and is time-consuming. To further assess athletes’ fatigue states, scholars have started using perceived exertion scales, the rating of perceived arousal scale, the sensation scale, the Hooper index, and SFMS questionnaires to detect signs of overtraining (Polito et al., 2017). Pillitteri, G. et al. (Pillitteri et al., 2023) used the RPE and TreS scales to assess players’ training loads, allowing coaches to better adjust and schedule training within microcycles to improve performance while reducing the risk of injury. However, scale results inevitably carry a considerable degree of subjectivity.

To more objectively assess athletes’ physiological fatigue, objective physiological markers such as heart rate and blood lactate levels measured by a heart monitor and lactate analyzer, respectively, can be used. Employing this objective indicator approach helps enhance the objectivity and accuracy of assessing athletes’ states. Lee, S. et al. (Lee et al., 2023) confirmed the role of lactate in exercise performance and further evaluated its impact on exercise training. Papacosta, E. et al. (Papacosta and Nassis, 2011) measured physiological biomarkers in whole saliva, providing a crucial tool for assessing immune and endocrine states related to exercise and training. However, these objective markers should be considered alongside subjective markers of training load, such as perceived exertion ratings, to more accurately quantify training loads and their perception (Djaoui et al., 2017).

## 3 Research methods and procedures

**Fig. 1:**
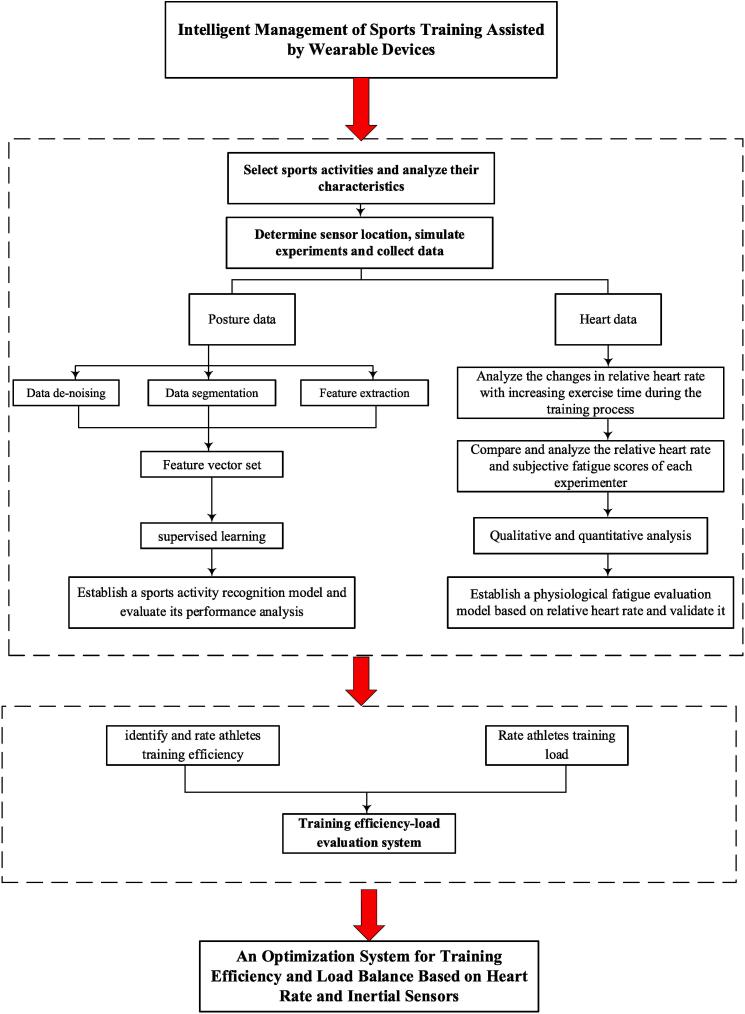
Training efficiency and load balancing optimization system.

Fig. 1 illustrates the schematic diagram of the system. Firstly, a sensor-based sports activity recognition model is constructed to validate its feasibility and conduct an analysis of athletes’ training efficiency. Next, the physiological fatigue during athlete training is explored. The main focus will be on establishing a physiological fatigue assessment model using two internal load indicators: heart rate and RPE (Rating of Perceived Exertion). This model aims to quantitatively analyze the training load on athletes. Finally, a balance optimization of training efficiency and exercise load is performed to guide athletes in effective training and scientifically managing physical loads. This section will provide a detailed explanation of the research methods and procedures used in the study. It includes (1) selection and analysis of sports activities, (2) collection and (3) processing of posture data, (4) establishment of the sports activity recognition model, and (5) collection of internal load data.

### 3.1 Sports activity selection and feature analysis

Shoulder injuries often occur in weightlifting athletes (Sim and Lie, 2023), and once a severe injury happens, it is challenging to return to sports. Moreover, weightlifting is a highly demanding sport (Ames et al., 2023), with weightlifters experiencing significant physical exhaustion and increased susceptibility to fatigue. Therefore, this study primarily focuses on weightlifting athletes as the research subjects. The weightlifting activity is divided into four key steps: gripping, lifting-off, clean and jerk, and setting-down. As the weightlifting in this study involves a professional and intricate sports activity, it encompasses a series of coherent movements. To gain a better understanding, we have provided a detailed breakdown of the movement steps outlined in Table S1, which is included in the supplementary materials.

After excluding the potential impact of sensor placement on motion, the study analyzed the characteristic changes in the left forearm, left wrist, left ankle, right forearm, right wrist, and right ankle of athletes during eight movements in training. These characteristic changes are reflected through the acceleration and angle variations of different body parts, with the most representative areas containing the characteristic information of each movement. Detailed results of the analysis of each movement’s characteristics are presented in Table S2.

From Table S2, it can be observed that the motion characteristics of the upper body are richer than those of the lower body during weightlifting, and the information at the wrist position is more abundant compared to the forearm. It is noteworthy that the use of two sensors, as opposed to more than two sensors, did not significantly decrease recognition accuracy. Additionally, the extensive collection of redundant data did not significantly enhance the accuracy of the activity recognition model; instead, it increased the burden of data processing. Therefore, this study chose to place inertial sensors on the right and left wrists to collect activity data from these two positions.

### 3.2 Collection of posture data

The experimental device used in this study is the WT901SDCL model. This device is capable of collecting three-axis acceleration and angular velocity data, with an acceleration range of up to ± 16 g and an angular velocity range of up to ± 2000°/s. Utilizing dynamic Kalman filtering algorithms and attitude solvers, the module can consistently output three-axis angles with an angle accuracy of 0.01°, meeting the requirements for data analysis. Moreover, the dimensions of this individual device are only 51.3 mm × 36 mm × 15 mm, making it convenient for carrying and fixing. Additionally, the device comes with an SD card for easy data storage. After the experiment, data can be exported to a computer for subsequent data processing and analysis by connecting to a PC host machine.

After determining the data collection device, the sampling frequency needs to be considered, balancing the accuracy of motion recognition and resource consumption. A high sampling frequency increases accuracy but also increases computation time, reducing practicality, while a low sampling rate leads to incomplete data, reducing the effectiveness of activity recognition. Therefore, an appropriate sampling rate should be chosen based on the type of research activity. Previous studies (Antonsson and Mann, 1985) have shown that the frequency of human daily activities ranges from 0 to 20 Hz, and the sampling frequencies used in related research vary from 2 to 125 Hz, all achieving satisfactory recognition accuracy. Therefore, in this study, considering relevant research and the characteristics of the activity, a sampling frequency of 50 Hz was chosen, meaning that data points are collected at a rate of 50 per second.

To establish a motion recognition model based on multiple types of inertial sensors, Motion Simulation Experiment 1 was designed to collect data from six healthy athletes performing four actions: gripping, lifting-off, clean and jerk, and setting down. All participants were students from the School of Physical Education, in good health without movement disorders or chronic fatigue. The basic information of the participants is shown in Table S3. Before participating in the study, all participants were informed of the goals, experimental procedures, and risks related to the study. All participants agreed to participate in this research.

Twenty minutes before the experiment, all participants arrived at the test location (Sports Training Center) and were briefed on the experimental procedure and precautions. Participants attached the inertial sensors to their left and right wrists using straps, started warming up in a natural posture and state, and tested whether the data collection devices were properly secured. After fixing the data collection devices, each participant performed the specified activities, including gripping, lifting-off, clean and jerk, and setting down the barbell. The experiment consisted of five sets to ensure sufficient data collection for subsequent algorithm training and testing. A recorder was assigned to use a camera to record the entire experiment process for later data processing and annotation. The collected activity data section is shown in the Fig. S1.

### 3.3 Preprocessing of inertial sensor data

The data collected by the sensors is raw continuous data that needs processing before it can be used for action classification and recognition. Data processing involves denoising, segmentation, and feature extraction, which is crucial for improving the accuracy of action recognition.

#### 3.3.1 Data de-noising

Due to the influence of human motion, environmental factors, and the method of data collection by wearable devices, the raw data collected often contains noise, which can degrade data quality. For instance, placing the sensor device casually in a pocket without proper fixation may result in unnecessary shaking. These interference data not only fail to accurately reflect human motion characteristics but also impact the accuracy of activity recognition. Therefore, it is necessary to apply appropriate denoising techniques to the data. Compared to non-fixed collection strategies that lead to accelerometer signal noise, collecting data with the sensor device securely fixed can reduce noise to some extent. This fixation strategy not only decreases the number of peaks, making the data flow smoother, but also improves the quality of the raw data. To minimize the impact of interference data, this experiment adopts a fixed collection strategy and does not apply additional denoising processing. The acceleration signal difference between fixed and non-fixed acquisition mode can be seen in Fig. S2.

#### 3.3.2 Data segmentation

Although data from various sport activities are included in continuous data streams, extracting features directly from these continuous data streams and performing action classification and recognition is extremely challenging. Therefore, the data signals need to be segmented. Data segmentation is typically achieved by applying sliding windows on the continuous data. Sliding windows can be classified into fixed-time windows and adaptive-time windows. The former has simpler preprocessing and a smaller model computational burden. Thus, this study chooses fixed-time windows.

In the design of sliding windows, they can be categorized as overlapping and non-overlapping time windows based on whether there is overlap between the windows. To maintain consistency in the segment size after data segmentation, facilitate feature extraction, and effectively distinguish between different types of activities, this study adopts a 50 % overlap rate. Considering the complexity of activities, model performance, and computational costs, the final approach uses fixed-size sliding overlapping windows for data segmentation. Past studies (Morales and Akopian, 2017) have shown that window lengths are usually between 1 and 7 s. Overly large window sizes may lead to different types of activity data being contained within the same window, reducing the accuracy of activity classification. Therefore, in this method, the window length is set to 5 s. Fig. S3 illustrates the concept of the fixed-size sliding overlapping windows.

#### 3.3.3 Feature extraction

The features extracted from human activity recognition based on wearable devices mainly include two categories: time domain features and frequency domain features. Compared with the frequency domain features with high computational cost, this study mainly selects the time domain features suitable for motion recognition with high real-time requirements. It includes: mean, standard deviation, interquartile range, kurtosis, skewness, covariance. By extracting these six eigenvalues, the attribute features of the four activities studied in this paper are raised from the original 12-dimensional to 72-dimensional, and the differences between activities will be more obvious. The feature vector C = [c_1_, c_2_, c_3_… c_72_] will be used as the feature vector set of each type of activity and input into the classifier for training for subsequent motion recognition models.

### 3.4 Classification and recognition of sports activities based on random forest algorithm

#### 3.4.1 Data label

Before inputting the feature vector into the classifier for training, corresponding data labels should be added to ensure that the sensor data of each time period corresponds to the sports activities it represents. This is a necessary step for supervised classification learning algorithms. Based on the video data captured by the experiment recorder, numerical labels ranging from 1 to 4 were assigned to the data in each time window according to the exercise steps.

#### 3.4.2 Construction and evaluation of the random forest algorithm classification model

After collecting raw data from sports activities and performing feature extraction, this study utilizes the random forest algorithm in machine learning to construct a classification model. MATLAB 2018a is employed as the programming environment for data processing, feature computation and extraction, model construction, validation, and evaluation. The covre code of the main program are contained in a separate GitHub repository (https://github.com/ManTang5/code-about-sports-activities-recognition-model).

The recognition of sports activities is not only related to the predictive classification capability of the random forest model but also to the quality of the raw data and feature extraction. After constructing the sports activity recognition model, it is essential to test the model’s performance and evaluate its accuracy in recognizing athlete activity information. Therefore, this study establishes a performance evaluation system for the RF model, including metrics such as “accuracy,” “precision,” “recall,” and “F1 score,” to assess the activity classification recognition performance of the RF model.

The code for the calculation rules of each evaluation metric can be seen in the GitHub repository. The application of the “confusion matrix” can also more intuitively reflect the model’s performance. For the selection of recognition data, a randomly generated 7:3 ratio of mutually exclusive training and testing sets is employed. The “hold-out” method is used for validation, conducting ten repeated experiments on the generated random forest model to obtain an average test result, thereby avoiding the interference of incidental results and ensuring the model’s authenticity.

The activity recognition model established in this study is based on collecting sample data from six participants working for several minutes. In the future, to further enhance the model’s supervision and management capabilities for athletes, the collected athlete activity data can be initially used as a test set for recognition. The real data from additional activities can then be added to the sample database after testing, serving as a training set. This continuous expansion of the sample database improves the total quantity and authenticity of the training set. With the ongoing expansion of data, the training model based on the random forest algorithm will continually improve. This will give the activity recognition model developed in this study higher generalization capabilities, allowing for increased accuracy in identifying new activity data.

### 3.5 Collection of internal load data

To facilitate participants in wearing devices during sports activities, this study procured heart rate monitoring devices with the model Fibit Charge 2. This device is a wrist-type heart rate measurement device (PPG-type heart rate sensor) capable of accurately recording users’ instantaneous heart rate data and storing it offline for convenient data processing and analysis. In addition, the study required participants to rate their perceived exertion using the Borg Rating of Perceived Exertion (RPE 6–20) scale to measure the level of fatigue during the exercise. The specific content of the RPE 6–20 scale is detailed in Table S4. Before the experiment, participants were thoroughly introduced to the distinctions and examples of each value on the RPE 6–20 scale to ensure that participants could provide corresponding RPE values based on their perceived exertion.

The purpose of this experiment is to collect data on the changes in heart rate and subjective fatigue during athletes’ training processes, providing a basis for the establishment and validation of the physiological fatigue assessment model. The participants are the same as those in Experiment 1, all of whom are physically and mentally healthy. To ensure the referenceability of experimental data, the heart rate levels during exercise will not fluctuate significantly due to pathological reasons. The specific experimental procedure is as follows:

(1) Before exercising, ensure that participants have not engaged in other physical activities, and their bodies and minds are in a completely relaxed state. Measure the heart rate levels of the six participants in a sitting position, with a time control of 5 min, using the average heart rate within 5 min as each participant’s resting heart rate (Hc).
(2) Conduct the experiment separately, requiring participants to wear heart rate monitoring devices and perform the specified weightlifting activities. The process consists of six sets, estimated to last 30 min, with specific activity content identical to Experiment 1. The standard barbell is used as the sports equipment, and participants choose the commonly used weight based on their daily training. An assigned person asks participants about their fatigue every 5 min and records the RPE score. Apart from this, no interference is introduced to ensure that participants are in a continuous exercise state, aligning with the conditions of regular sports training.
(3) After each participant completes the activity, import the heart rate data to the computer for processing. The average heart rate per minute is used to represent the participant’s instantaneous heart rate (Hw) during exercise.

## 4 Data analysis and interpretation

This section will analyze the experimental results. Firstly, the performance of the sport activity recognition model will be examined. Through the established performance evaluation system for the RF model, various indicators of the model will be computed to assess its performance in classifying construction activities. Subsequently, each construction activity will be categorized, and an assessment method for training efficiency based on “effective work rate” will be established, providing data reference for professional management. Secondly, a physiological fatigue assessment model based on relative heart rate will be developed to qualitatively and quantitatively analyze the physiological fatigue of athletes. Finally, incorporating auxiliary tools such as effective training rate and time proportion charts, a balanced optimization system for training efficiency and load will be developed to evaluate the efficiency-load levels of athletes.

### 4.1 Performance analysis of the random forest classification model

Due to individual differences, it is necessary to discuss the differences in individual data samples and sample sets during the classification performance testing, ensur that the classification model can adapt to various complex sports activities. In the classification training phase, training is initially conducted on the six individual data samples. Subsequently, these six individual data samples are combined to form a sample set, and training is performed on the sample set. In the model validation phase, the study employs the “holdout method” with a training set to test set ratio of 7:3. This process is repeated ten times, with a redivision of the training set and test set in each iteration.

The classification test results for individual data samples are shown in Table 1. The random forest model achieved over 90 % accuracy for the four activities performed by the six experimental participants, with an average classification accuracy of 94.24 %. This indicates that various complex sports activities can be classified using the classification model established in this study. Similarly, the classification test results for the sample set are shown in Table S5. The table indicates an average classification accuracy of 89.94 % for the sample set, which is a 4.3 % decrease compared to the model’s average classification accuracy of 94.24 % for individual samples. Clearly, the inclusion of activity data from different individuals in the sample set introduces diversity and complexity due to individual variations, leading to a reduction in classification accuracy. However, as discussed in Section 3.4, an expanding database will enhance the authenticity of the training set, thereby improving the model’s generalization ability. This will play a better predictive role for new data sets, a point that will be further confirmed in Section 4.2.

**Table 1:** Classification test results of individual samples.

Experimental personnel	Classification accuracy (%)										
	1	2	3	4	5	6	7	8	9	10	mean value
1	91.24	93.82	92.91	92.03	90.34	91.22	92.01	92.93	92.13	93.42	92.21
2	97.34	96.53	98.23	92.92	94.69	92.04	93.81	95.58	93.81	92.04	94.70
3	95.58	98.23	94.78	96.46	97.35	96.54	94.74	95.69	96.38	94.67	96.04
4	92.92	95.56	92.04	91.15	93.27	91.36	92.46	95.58	93.25	90.27	92.79
5	98.23	97.45	96.74	95.78	95.48	95.69	96.93	97.36	97.26	96.73	96.77
6	94.69	93.81	92.92	94.37	93.69	90.27	92.04	91.06	92.84	93.58	92.93
mean value	–	–	–	–	–	–	–	–	–	–	94.24

### 4.2 Performance analysis of sports activity recognition based on random forest classification model

After verifying the authenticity of the model’s recognition results, we conducted validation on the model’s predictive classification ability for unknown data. Starting with sample data 1, we gradually expanded the training database, including samples 2–6, and conducted model training and prediction classification each time. For each data set, the model was trained. For example, using the combination of sample data 1 and 2 as database 2 for model training, then predicting the classification for samples 3–6, and so on, until using the combination of sample data 1–5 as database 5 for model training. The prediction test results for each database are shown in Table S6.

Taking the optimal model trained based on database 5 as an example, Table 2 presents the predicted results for the four sports activities, including precision, recall, and F1 score. As shown in the table, the precision distribution range is 0.9091 to 1, the recall distribution range is 0.8750 to 1, and the F1 score distribution range is 0.9130 to 1. Precision and recall judge the model’s performance from different perspectives, as can be well understood from the calculation formulas. However, high or low precision and recall alone cannot comprehensively reflect the model’s performance. Therefore, it is essential to continue analyzing the F1 score. The F1 scores for each activity are all above 0.9, indicating the model’s strong recognition capability for various weightlifting activities.

**Table 2:** Prediction results of sports activities.

Training Activities	Precision	Recall	F1 Score
Grip	0.9545	0.8750	0.9130
Lift-off	0.9375	0.9677	0.9524
Clean and Jerk	0.9091	0.9375	0.9231
Set-down	1	1	1

**Fig. 2:**
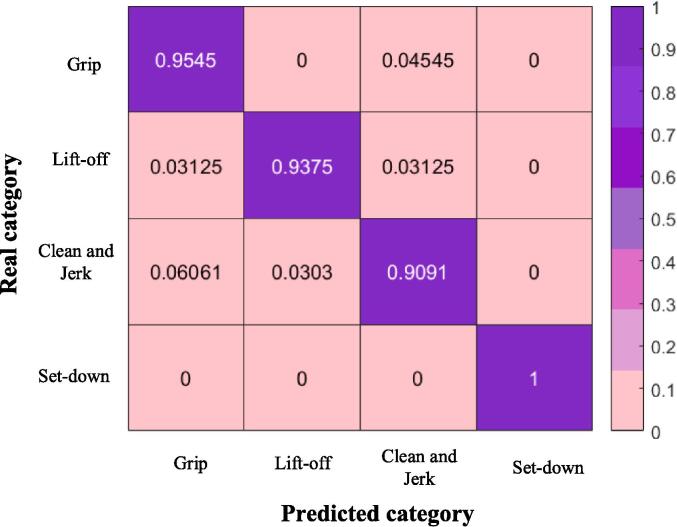
Confusion Matrix for Weightlifting Activities.

Furthermore, the performance of the model is visually analyzed using a confusion matrix. The confusion matrix provides a more intuitive representation of the relationships between different activities. Fig. 2 displays the confusion matrix based on database 5. The activity of “grip” shows extremely high distinctiveness, achieving a prediction precision of 100 %. In contrast, the prediction precision for “clean and jerk” is the lowest, at only 90.91 %, and it is mistakenly predicted as “lift-off”. This is because, during weightlifting training, there is some similarity between the activity data generated during “lift-off” and “clean and jerk”. However, an overall prediction precision of over 90 % is sufficient to meet the requirements of recognizing complex sports activities in real-time and can contribute to real-time recognition of athletes’ activities.

### 4.3 Analysis of athlete training efficiency

Based on the above, this study divides the four types of sports activities into three categories: effective training, auxiliary training, and non training, and calculates the homework time for each activity type. Introduce the evaluation indicator of “effective training rate” and evaluate the training efficiency of athletes using a time ratio chart, providing data support and management suggestions for coaches.

(1) Effective training rate

Based on the characteristics of weightlifting activities, lifting-off and clean&jerk are classified as effective training training, while gripping and setting-down are categorized separately as auxiliary training and non-training movements, respectively. By utilizing the collected data samples, the time spent on each activity can be determined. The effective training rate is defined as the ratio of effective training time to total training time, with the formula:

(1) $$\theta =\;T_{e}/T$$

(Note: θ Represents effective training rate, te represents effective training time, and T represents total training time.)

According to the formula, when θ When it is higher, it indicates that the training efficiency of the athlete is higher; When θ When the training efficiency is low, it indicates that the training efficiency is low. Coaches should pay attention to whether there are slacking off or athletes experiencing training injuries in the training room.

(2) Time scale chart

**Fig. 3:**
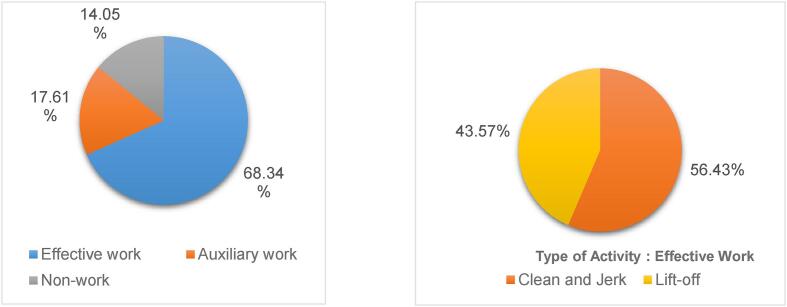
The time proportion of each weightlifting activity.

On the basis of achieving high-precision recognition of athletes’ various activities, taking the activity data of Experiment 6 as an example, a time proportion chart of sports activities was established based on the types of sports activities and their time consumption, as shown in Fig. 3.

### 4.4 Evaluation of Athletes’ physiological fatigue based on relative heart rate changes

#### 4.4.1 Rating of perceived exertion (RPE)

The subjective fatigue perception (RPE scores) of the six participants during the weightlifting task is depicted as Fig. S4. Comprehensive analysis reveals an overall positive correlation between RPE scores and exercise duration, but significant individual differences exist. Despite using a constant exercise speed in the experiment, these individual differences lead to varying training intensities for each athlete. For some participants, subjective fatigue scores remain relatively stable, indicating that the training intensity of the weightlifting task designed in the experiment is appropriate. Conversely, for other participants, subjective fatigue scores significantly increase with the duration of exercise, suggesting a relatively high training intensity in the experiment’s design.

The RPE scale to some extent reflects the individual differences among athletes. However, in practice, this method is not only time-consuming but also has a certain degree of lag. Additionally, athletes are influenced by various factors such as exercise emotions, social approval, and differences in understanding the grading of RPE. As a result, when expressing their subjective feelings of fatigue, they may exhibit some bias. Therefore, it is crucial to combine more objective physiological indicators for real-time assessment to comprehensively understand the physiological fatigue situation of athletes.

#### 4.4.2 Relative heart rate

Considering the individual differences in resting heart rate levels, we introduced the more scientifically grounded physiological indicator of relative heart rate to assess the physiological fatigue of athletes during training. Relative heart rate refers to the ratio of instantaneous heart rate to resting heart rate during exercise, as follows:

(2) Hr=Hw/Hc

The relative heart rate values for each participant in the weightlifting exercise are shown in Fig. S5. We observed a continuous upward trend in relative heart rate during the exercise process.

In the initial stage of exercise (3–5 min), the heart rate of each participant quickly rose to 1.21–1.42 times their resting heart rate, with heart rate ranging from 91 to 104 beats/min, indicating adaptation to the training intensity. Between 5 and 20 min, some participants had only slight fluctuations in relative heart rate, while others showed a significant increase, indicating different levels of physiological fatigue. At 30 min, the relative heart rates of each participant continued to fluctuate upward, measuring 1.54, 1.52, 1.64, 1.58, 1.53, and 1.46, respectively.

Relative heart rate, compared to subjective fatigue scores, more objectively illustrates the physiological fatigue changes of participants during exercise. For example, participant 6 had an RPE score of 13 at 30 min, higher than participants 1, 2, and 4. This implies that if only relying on the subjective fatigue quantification table to judge the fatigue state of athletes, participant 6 would be considered more fatigued than the other three participants. However, after analyzing heart rate and relative heart rate data, it can be observed that participant 6′s heart rate level was only 114 beats/min, and the relative heart rate of 1.46 was lower than the other three participants. This indicates that from an objective physiological perspective, the actual physiological fatigue level of participant 6 is lower than that of participants 1, 2, and 4.

#### 4.4.3 Physiological fatigue evaluation model based on relative heart rate

In order to eliminate the impact of individual differences on the model, this study employed two evaluation indicators: average relative heart rate and average RPE score, and conducted nonlinear fitting. The fitting results are shown in Fig. 4a, Fig. 4b, with corrected determination coefficients (R^2^) of 0.927 and 0.982, indicating an overall good fitting effect. Relative heart rate and subjective fatigue scores are closely related, exhibiting similar changing trends with increasing exercise duration, validating the feasibility of physiological fatigue assessment based on relative heart rate.

**Fig. 4a:**
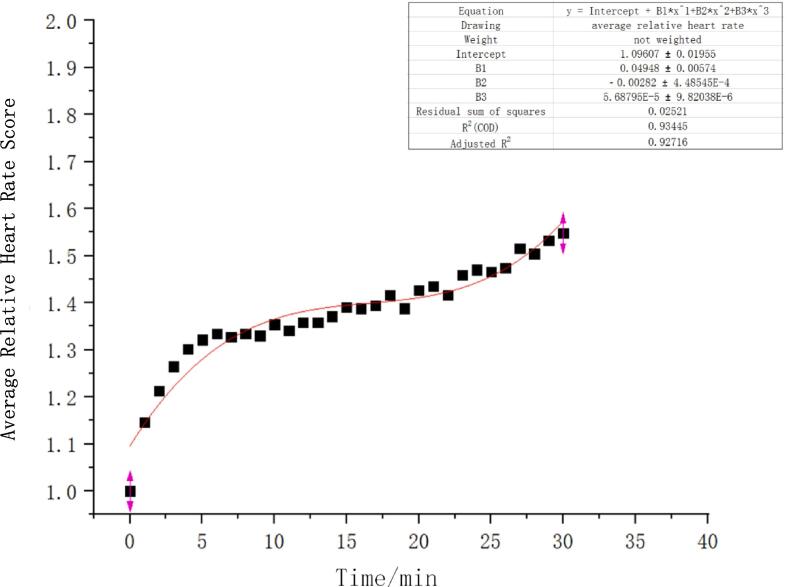
Average relative heart rate over time.

**Fig. 4b:**
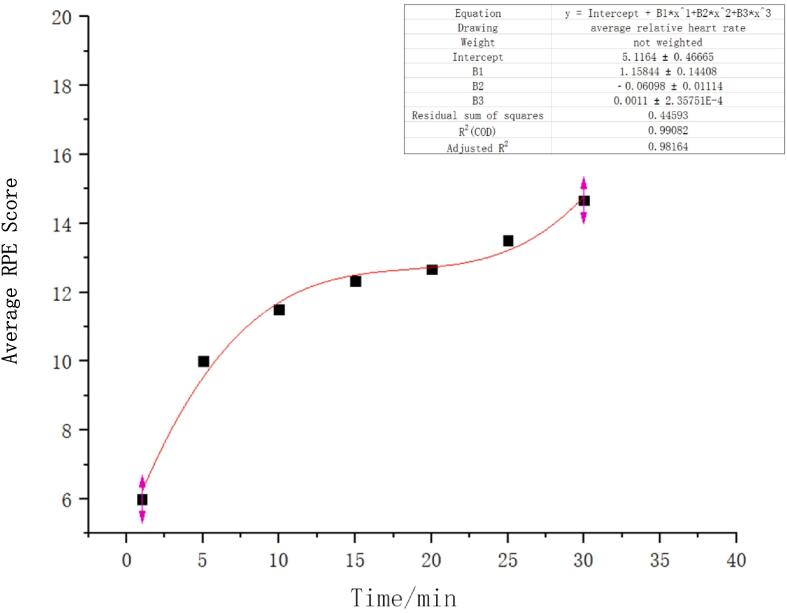
Average RPE score over time.

Based on the relationship between relative heart rate and subjective fatigue scores, a physiological fatigue assessment model was established, expressed as: y = 16.12343x − 10.36787. Model testing yielded a corrected determination coefficient (R2) of 0.973 and an F-test score of 219.73, indicating a good overall fitting effect. The fitting results are depicted in Fig. 4(c). This model enables qualitative and quantitative analysis of athletes’ physiological fatigue. When applying the model, real-time heart rate is measured using a portable heart rate monitoring device, the relative heart rate (x-value) is calculated, and the model equation is then used to predict the y-value, representing the actual fatigue intensity. For example, considering the data for participant 3 at 25 min of exercise, the calculated relative heart rate value is 1.469, and the model predicts an actual fatigue intensity of 13.3, indicating a fatigue level between slight fatigue and fatigue. When the predicted RPE value exceeds 20, it suggests that the athlete is undergoing a significantly intense training, with a notable increase in safety risks.

**Fig. 4c:**
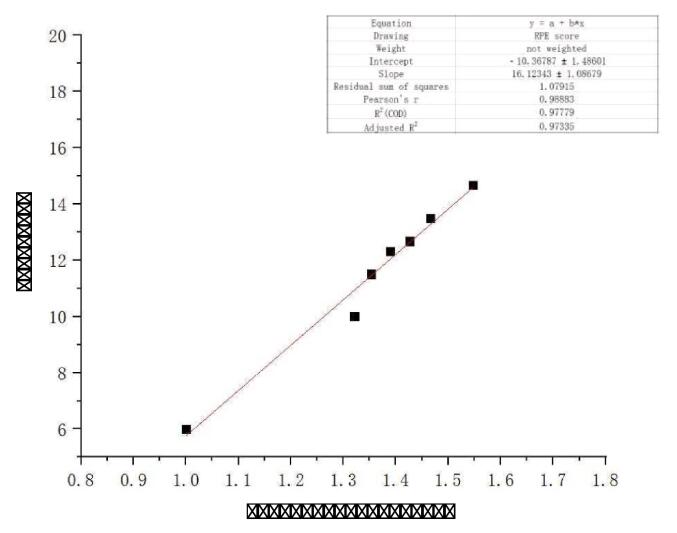
Fitting diagram of relative heart rate - RPE score.

The physiological fatigue assessment model based on relative heart rate, combined with subjective and objective indicators, provides coaches with a scientific tool for real-time monitoring of athletes’ physiological conditions and fatigue levels. This contributes to enhancing the safety management level and efficiency of sports training, possessing significant theoretical and practical value.

### 4.5 Training ffficiency and load balance optimization system

After verifying the scientificity and accuracy of evaluating the physiological fatigue level of athletes based on relative heart rate, combined with the physiological fatigue evaluation model of relative heart rate and the RPE values corresponding to different relative heart rates, the training load of the experimental personnel was divided into three levels: low (<1.33), medium (1.33–1.57), and high (>1.57).

The sports recognition model based on wearable devices showed extremely high prediction accuracy in Experiment 1, and can effectively identify and classify various weightlifting activities. By combining this model with the physiological fatigue evaluation model, the training efficiency of athletes is evaluated through a proportional graph of effective training rate and activity time. According to the data results of Experiment 1, the training efficiency is divided into three levels: low (<35 %), medium (35 %-55 %), and high (>55 %).

Taking into account training load and efficiency rating methods, an efficiency load evaluation system is established as shown in Table 3, which divides athlete states into nine types. This evaluation system not only reflects the comprehensive sports status of athletes, but also reveals the problems in training and provides management suggestions for coaches.

**Table 3:** Efficiency-load evaluation system for athletes.

	Low efficiency	Medium efficiency	High efficiency
LOW LOAD	There may be situations in which the athlete is not serious or just to deal with the training. It is recommended to urge the training to be carried out normally.	The more ideal training state	Ideal training state, it is recommended to study its training methods and promote
MWDIUM LOAD	The effect is poor, which may be related to the training method or the layout of the training ground. It is recommended to replace the training program.	The ideal training state	The more ideal training state
HIGH LOAD	Athletes may have physiological hidden dangers, it is recommended to check the record in a timely manner	There may be room for improvement in the training method, or the physical condition on the day is general, and appropriate rest should be paid attention to.	It is recommended to control the length of training, take appropriate rest, and beware of excessive fatigue.

**Fig. 5:**
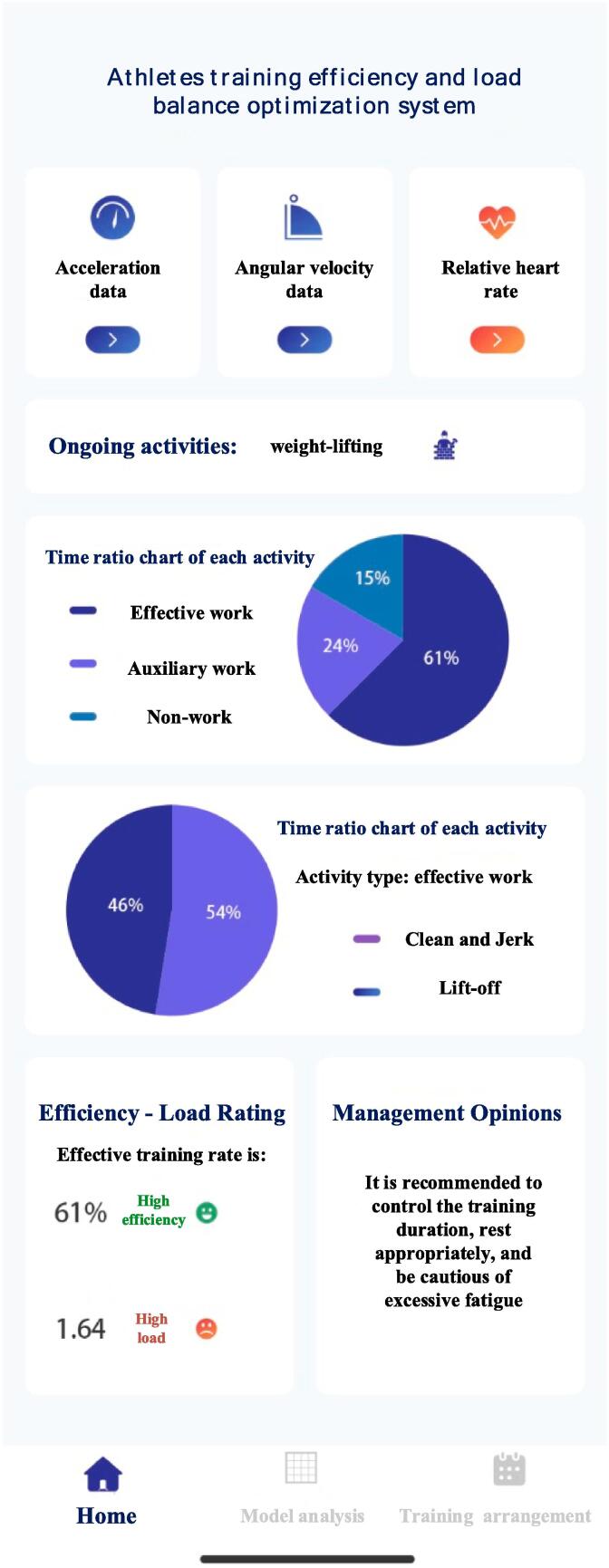
Main interface of training efficiency and load balance optimization system for athletes.

Furthermore, a sports activity recognition model and a physiological fatigue evaluation model were combined to develop an optimized system for balancing exercise workload based on the fusion of heart rate and inertial sensors. The system collects heart rate, acceleration, and angular velocity data through sensors. After activity recognition and physiological fatigue evaluation, the comprehensive exercise status of athletes is ultimately rated based on the ergonomic load evaluation system. The system interface is shown in Fig. 5, providing intuitive comprehensive motion status and management suggestions.

## 5 Discussion

This section will further discuss two aspects: (1) accuracy of the system and (2) practical application.

### 5.1 Accuracy of the system

In this study, the excellent performance of various models provides the system with scientific validity and accuracy. The sports activity classification model achieved a high prediction accuracy of 94.70 %, with F1 scores above 0.9 for all four complex weightlifting activities and reaching 1 for the “snatch” activity. This demonstrates the overall outstanding performance of the model developed in this study, enabling precise recognition of complex activities. The activity recognition model, based on the random forest machine learning method, exhibited good accuracy during training. It achieved high accuracy rates of 94.24 % and 89.94 % on individual sample databases and sample aggregate databases, respectively. As a supervised learning tool for activity classification, we do not exclude the possibility that other machine learning methods may have superior performance in classification, so we have not restricted the use of other machine learning methods in this manuscript. Additionally, the physiological fatigue model verification yielded favorable fitting results. The adjusted coefficient of determination (R2) was 0.973, with an F-test score of 219.73.

### 5.2 Practical application

Firstly, despite the recommendation to assess both internal and external training load parameters for insights into training stress (Halson, 2014), there is currently no standardized direct coupling of internal and external training loads (van der Zwaard et al., 2023). Using weightlifting athlete training data as an example, this study quantifies athletes’ external load and analyzes their training duration. Subsequently, a physiological fatigue model is established by combining internal loads measured by RPE and relative heart rate, categorizing athletes’ training states into nine types. This information is visualized through a system interface to enhance the management of specific training courses. Secondly, this study introduces the metric “Effective Training Rate”. It measures the actual training effects achieved by athletes during the training process, providing a crucial metric for assessing athletes’ adaptability and training effectiveness. Through a systematic assessment of training efficiency, we can gain a clearer understanding of the impact of different training activities on athletes’ load and adaptability, offering a scientific basis for personalized training programs. Finally, in comparison to the complex and highly specialized TRIMP score internal load monitoring method (Halson, 2014), this study employs the RPE and relative heart rate monitoring methods. This choice not only reduces the operational complexity of monitoring but also aligns more with the practical requirements of training scenarios. The application of RPE and relative heart rate provides a more flexible and easily implementable method for internal load assessment, making training monitoring more closely aligned with athletes’ actual experiences. Moreover, recent research demonstrates the effectiveness and reliability of RPE in full-body strength and endurance training (van der Zwaard et al., 2023). Combined with the objective measure of relative heart rate, this further enhances the reliability of physiological fatigue assessment.

## 6 Conclusion

This study utilized wearable devices, incorporating heart rate monitors and inertial sensors, to monitor athletes’ physiological fatigue. It developed a Sports Performance and Workload Balance Optimization System, providing insights into athletes’ physical health and fatigue levels. Through the collection of weightlifting data using various types of inertial sensors, an efficient activity recognition model was established, demonstrating outstanding performance in terms of classification accuracy. Building upon this foundation, the study achieved the management of athletes’ training efficiency, classification, and the establishment of a rating method. The research also assessed athletes’ physiological fatigue through relative heart rate, validating the scientific and precise nature of this physiological indicator. A physiological fatigue assessment model based on relative heart rate was established, leading to the creation of an athlete training workload rating method. Finally, in conjunction with training efficiency ratings, an athlete’s performance-load evaluation system was established, categorizing the athlete’s exercise state into nine comprehensive assessments.

However, several limitations exist in this study. Firstly, the participant sample size is limited to only six individuals, restricting the reliability of the research. Future studies should aim to increase the sample size. Secondly, due to individual differences and the lack of a gold standard for body fatigue, there are certain limitations in the definition and measurement of physical fatigue. Lastly, future research could explore more accurate fatigue assessment tools and advanced physiological measurement devices.

## CRediT authorship contribution statement

**Chen Wang:** Writing – original draft. **Man Tang:** Writing – original draft. **Kun Xiao:** Writing – review & editing. **Defa Wang:** Funding acquisition. **Bin Li:** Funding acquisition.

## Declaration of competing interest

The authors declare the following financial interests/personal relationships which may be considered as potential competing interests: This research received funding from the Fujian Provincial Department of Science and Technology (Grant Number: 2021I0014) and the Xiamen Municipal Construction Bureau (Grant Number: XJK2022-1-7). There are no potential financial conflicts of interest among the authors. We declare that we have not been influenced by any commercial interests and have not received any other forms of financial support to influence the outcomes of this research. The authors of this study have no affiliations with any commercial entities that might have an impact on the research results. We ensure that there have been no influences or interference from any commercial partners throughout the course of this study.

## Data Availability

Data will be made available on request.

## References

[b0005] Alghamdi W.Y. (2023). A novel deep learning method for predicting athletes’ health using wearable sensors and recurrent neural networks. Decision Analytics Journal.

[b0010] Ames A., Shah S.S., Pettit R., Li L., Chilton M., Gaylord B., Ross G. (2023). Against surgeons’ advice: the return to sport in high-demand weightlifters following anatomic total shoulder arthroplasty at average 3.6 years’ follow-up. J. Shoulder Elbow Surg..

[b0015] Antonsson E.K., Mann R.W. (1985). The frequency content of gait. J. Biomech..

[b0020] Bompa, T. O., & Haff, G. G. (2009). Periodization. Theory and methodology of training, 5.

[b0025] Catal C., Tufekci S., Pirmit E., Kocabag G. (2015). On the use of ensemble of classifiers for accelerometer-based activity recognition. Appl. Soft Comput..

[b0030] Chen Y., Wang Z. (2017). A hierarchical method for human concurrent activity recognition using miniature inertial sensors. Sens. Rev..

[b0035] Cheng W.C., Jhan D.M. (2012). Triaxial accelerometer-based fall detection method using a self-constructing cascade-AdaBoost-SVM classifier. IEEE J. Biomed. Health Inform..

[b0040] Cui J., Du H., Wu X. (2023). Data analysis of physical recovery and injury prevention in sports teaching based on wearable devices. Prev. Med..

[b0045] Djaoui L., Haddad M., Chamari K., Dellal A. (2017). Monitoring training load and fatigue in soccer players with physiological markers. Physiol. Behav..

[b0050] Fang Y.M., Chang C.C. (2016). Users’ psychological perception and perceived readability of wearable devices for elderly people. Behav. Inform. Technol..

[b0055] Feng W., Zeng K., Zeng X., Chen J., Peng H., Hu B., Liu G. (2023). Predicting physical fatigue in athletes in rope skipping training using ECG signals. Biomed. Signal Process. Control.

[b0060] Gu F., Khoshelham K., Valaee S., Shang J., Zhang R. (2018). Locomotion activity recognition using stacked denoising autoencoders. IEEE Internet Things J..

[b0065] Halson S.L. (2014). Monitoring training load to understand fatigue in athletes. Sports Med..

[b0070] Impellizzeri F.M., Marcora S.M., Coutts A.J. (2019). Internal and external training load: 15 years on. Int J Sports Physiol Perform.

[b0075] Jobson S.A., Passfield L., Atkinson G., Barton G., Scarf P. (2009). The analysis and utilization of cycling training data. Sports Med..

[b0080] Khan Y., Ostfeld A.E., Lochner C.M., Pierre A., Arias A.C. (2016). Monitoring of vital signs with flexible and wearable medical devices. Adv. Mater..

[b0085] Lee, S., Choi, Y., Jeong, E., Park, J., Kim, J., Tanaka, M., & Choi, J. (2023). Physiological significance of elevated levels of lactate by exercise training in the brain and body. Journal of bioscience and bioengineering. 10.1016/j.jbiosc.2022.12.001 36681523

[b0090] Liu L., Qiu S., Wang Z., Li J., Wang J. (2020). Canoeing motion tracking and analysis via multi-sensors fusion. Sensors.

[b0095] Lyons G.M., Culhane K.M., Hilton D., Grace P.A., Lyons D. (2005). A description of an accelerometer-based mobility monitoring technique. Med. Eng. Phys..

[b0100] Maciejczyk M., Wiecek M., Szymura J., Cempla J., Wiecha S., Szygula Z., Brown L.E. (2014). Effect of body composition on respiratory compensation point during an incremental test. J. Strength Cond. Res..

[b0105] Mathie M.J., Coster A.C.F., Lovell N.H., Celler B.G. (2003). Detection of daily physical activities using a triaxial accelerometer. Med. Biol. Eng. Compu..

[b0110] Memar S., Delrobaei M., Gilmore G., McIsaac K., Jog M. (2017). Segmentation and detection of physical activities during a sitting task in Parkinson’s disease participants using multiple inertial sensors. J. Appl. Biomed..

[b0115] Memar S., Delrobaei M., Pieterman M., McIsaac K., Jog M. (2018). Quantification of whole-body bradykinesia in Parkinson’s disease participants using multiple inertial sensors. J. Neurol. Sci..

[b0120] Morales J., Akopian D. (2017). Physical activity recognition by smartphones, a survey. Biocybernetics and Biomedical Engineering.

[b0125] Ni Q., Fan Z., Zhang L., Nugent C.D., Cleland I., Zhang Y., Zhou N. (2020). Leveraging wearable sensors for human daily activity recognition with stacked denoising autoencoders. Sensors.

[b0130] Ohnishi A., Murao K., Terada T., Tsukamoto M. (2019). A method for structuring meeting logs using wearable sensors. Internet of Things.

[b0135] Papacosta E., Nassis G.P. (2011). Saliva as a tool for monitoring steroid, peptide and immune markers in sport and exercise science. J. Sci. Med. Sport.

[b0140] Pillitteri G., Rossi A., Simonelli C., Leale I., Giustino V., Battaglia G. (2023). Association between internal load responses and recovery ability in U19 professional soccer players: a machine learning approach. Heliyon.

[b0145] Polito L.F.T., Figueira A.J., Miranda M.L.J., Chtourou H., Miranda J.M., Brandão M.R.F. (2017). Psychophysiological indicators of fatigue in soccer players: a systematic review. Sci. Sports.

[b0150] Qamar N., Siddiqui N., Ehatisham-ul-Haq M., Azam M.A., Naeem U. (2020). An approach towards position-independent human activity recognition model based on wearable accelerometer sensor. Procedia Comput. Sci..

[b0155] Qiu S., Zhao H., Jiang N., Wang Z., Liu L., An Y., Fortino G. (2022). Multi-sensor information fusion based on machine learning for real applications in human activity recognition: state-of-the-art and research challenges. Information Fusion.

[b0160] Ride J., Ringuet C., Rowlands D., Lee J., James D. (2013). A sports technology needs assessment for performance monitoring in swimming. Procedia Eng..

[b0165] Serrano J.I., Lambrecht S., del Castillo M.D., Romero J.P., Benito-León J., Rocon E. (2017). Identification of activities of daily living in tremorous patients using inertial sensors. Expert Syst. Appl..

[b0170] Sim D.S., Lie D.T.T. (2023). Suprascapular nerve compression by spinoglenoid cysts arising from posterior labral tears: unusual presentation in young male gym enthusiasts–case report. Journal of Orthopaedic Reports.

[b0175] Stamm A., Thiel D.V., Burkett B., James D.A. (2011). Towards determining absolute velocity of freestyle swimming using 3-axis accelerometers. Procedia Eng..

[b0180] Suh S., Rey V.F., Lukowicz P. (2023). TASKED: Transformer-based Adversarial learning for human activity recognition using wearable sensors via self-KnowledgE distillation. Knowl.-Based Syst..

[b0185] Taylor K., Chapman D., Cronin J., Newton M.J., Gill N. (2012). Fatigue monitoring in high performance sport: a survey of current trends. J Aust Strength Cond.

[b0190] Thorpe R.T., Atkinson G., Drust B., Gregson W. (2017). Monitoring fatigue status in elite team-sport athletes: implications for practice. Int. J. Sports Physiol. Perform..

[b0195] Twist C., Highton J. (2013). Monitoring fatigue and recovery in rugby league players. Int. J. Sports Physiol. Perform..

[b0200] Uddin M.Z. (2019). A wearable sensor-based activity prediction system to facilitate edge computing in smart healthcare system. J. Parallel Distrib. Comput..

[b0205] Vacher P., Filaire E., Mourot L., Nicolas M. (2019). Stress and recovery in sports: effects on heart rate variability, cortisol, and subjective experience. Int. J. Psychophysiol..

[b0210] van der Zwaard S., Graafland F.H., van Middelkoop C., Lintmeijer L.L. (2023). Validity and reliability of facial rating of perceived exertion scales for training load monitoring. J. Strength Cond. Res..

[b0215] van der Zwaard S., Otter R.T., Kempe M., Knobbe A., Stoter I.K. (2023). Capturing the complex relationship between internal and external training load: a data-driven approach. Int. J. Sports Physiol. Perform..

[b0220] Wallace L.K., Slattery K.M., Coutts A.J. (2009). The ecological validity and application of the session-RPE method for quantifying training loads in swimming. J. Strength Cond. Res..

[b0225] Wang A., Chen G., Yang J., Zhao S., Chang C.Y. (2016). A comparative study on human activity recognition using inertial sensors in a smartphone. IEEE Sens. J..

[b0230] Wang, C. (2022). Sports-induced fatigue recovery of competitive aerobics athletes based on health monitoring. Computational Intelligence and Neuroscience, 2022. 10.1155/2023/9848053 PMC10631874 37946863

[b0235] Wilk M.P., Walsh M., O’Flynn B. (2020). Multimodal sensor fusion for low-power wearable human motion tracking systems in sports applications. IEEE Sens. J..

[b0240] Zhu Z., Li H., Xiao J., Xu W., Huang M.C. (2022). A fitness training optimization system based on heart rate prediction under different activities. Methods.

