# Supplementary material for: Optimization system for training efficiency and load balance based on the fusion of heart rate and inertial sensors
Source: Prev Med Rep. 2024 Mar 29;41:102710. doi: 10.1016/j.pmedr.2024.102710 (PMC10990899; doi:10.1016/j.pmedr.2024.102710)
Supplement: Supplementary data 1 [file mmc1.docx]

Table S1 Motion activity decomposition

| Activity types | Human body movements involved |
| --- | --- |
| Grip | Stand, stoop, grab the barbell, get up |
| Lift-off | Twist wrist, swing arms, raise arms |
| Clean and Jerk | Twist wrists and raise arms |
| Set-down | Bend, place the barbell, get up |

Table S2 Action characteristics analysis

| Action Types | Parts of body | | | | | |
| --- | --- | --- | --- | --- | --- | --- |
|  | Left lower arm | Left wrist | Left ankle | Right lower arm | Right wrist | Right ankle |
| Stand | - | - | - | - | - | - |
| Bend | ∆𝑎_d_ | ∆𝑎_d_ | - | ∆𝑎_d_ | ∆𝑎_d_ | - |
| Raise arms | ∆𝑎_u_ | ∆𝑎_ud_  ∆𝑔 | - | ∆𝑎_u_ | ∆𝑎_ud_  ∆𝑔 | - |
| Grab the barbell | ∆𝑎_dl_ | ∆𝑎_dl_  ∆𝑔 | - | ∆𝑎_l_ | ∆𝑎_ul_  ∆𝑔 | - |
| Get up | ∆𝑎_u_ | ∆𝑎_u_ | - | ∆𝑎_u_ | ∆𝑎_u_ | - |
| Place the barbell | ∆𝑎_dr_ | ∆𝑎_dr_  ∆𝑔_fb_ | ∆𝑔_fb_ | ∆𝑎_r_ | ∆𝑎_r_  ∆𝑔 | ∆𝑔_fb_ |
| Swing arms | ∆𝑎_u_  ∆𝑔_u_ | ∆𝑎_d_  ∆𝑔_lr_ | ∆𝑔_fb_ | ∆𝑎_r_  ∆𝑔_lr_ | ∆𝑎_u_  ∆𝑔_u_ | ∆𝑔_fb_ |
| Twist wrists | - | ∆𝑎_lrud_ | - | ∆𝑔 | ∆𝑎_lrud_  ∆𝑔 | - |
| Note: ∆ a: Changes in the acceleration of the body parts when completing each action  ∆g: Change of angle  “u, d, l, r, f, b”: Each corner represents the direction of change, including up, down, left, right, forward and backward  “- ”: There is no change in the acceleration and angle of the body part during the action. | | | | | | |

| Number | Gender | Height(cm) | Weight (kg) | Age | College | Physical quality |
| --- | --- | --- | --- | --- | --- | --- |
| 1 | male | 173 | 72.4 | 24 | physical culture institute | good |
| 2 | male | 178 | 74.6 | 26 | physical culture institute | good |
| 3 | male | 186 | 65.3 | 25 | physical culture institute | good |
| 4 | male | 174 | 76.5 | 26 | physical culture institute | good |
| 5 | male | 175 | 62.8 | 25 | physical culture institute | good |
| 6 | male | 176 | 80.2 | 25 | physical culture institute | good |
| Mean value | - | 177 | 71.9 | 25.2 | - | - |

Table S3 Experimental personnel information table


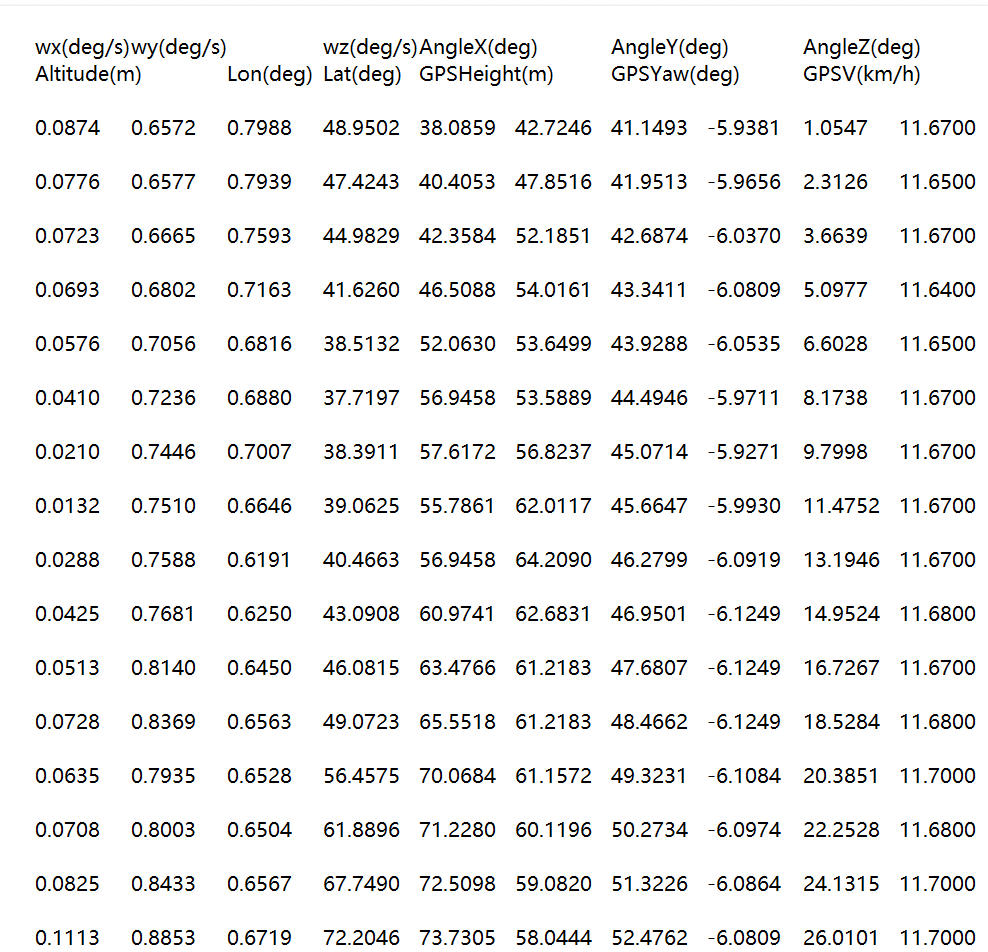


Fig.S1 Activity data schematic diagram of the left wrist

|  |  |
| --- | --- |
| Fig.S2 Difference in acceleration signal before and after  (Left is fixed acquisition mode, right is non-fixed acquisition mode)    Fig.S3 Schematic diagram of fixed-size sliding overlap window method | |

Table S4 RPE6-20 table

| RPE value | Subjective feelings | RPE value | Subjective feelings | RPE value | Subjective feelings |
| --- | --- | --- | --- | --- | --- |
| 6 | No feelings | 11 | relaxed | 16 | / |
| 7 | Extremely relaxed | 12 | / | 17 | Very tired |
| 8 | / | 13 | A little tired | 18 | / |
| 9 | Very relaxed | 14 | / | 19 | Extremly tired |
| 10 | / | 15 | tired | 20 | Give out |

Table S5 Classification test results of sample set

| Number of verifications | Classification accuracy (%) |
| --- | --- |
| 1 | 90.28 |
| 2 | 90.64 |
| 3 | 90.27 |
| 4 | 89.38 |
| 5 | 93.81 |
| 6 | 90.76 |
| 7 | 90.91 |
| 8 | 88.74 |
| 9 | 87.88 |
| 10 | 86.73 |
| mean value | 89.94 |

Table S6 The prediction test results for each database

| Database  Experimental personnel | 1 | 2 | 3 | 4 | 5 |
| --- | --- | --- | --- | --- | --- |
| 1 |  |  |  |  |  |
| 2 | 87.21 |  |  |  |  |
| 3 | 86.84 | 89.77 |  |  |  |
| 4 | 85.43 | 86.54 | 88.46 |  |  |
| 5 | 88.58 | 90.12 | 92.36 | 94.36 |  |
| 6 | 86.52 | 87.29 | 87.73 | 90.15 | 94.7 |
| Average prediction accuracy（%） | 86.92 | 88.43 | 89.52 | 92.26 | 94.7 |

Fig.S4 Curve of RPE score changing with time

Fig.S5 Curve of relative heart rate changing with time
